# The cerebellum is causally involved in episodic memory under aging

**DOI:** 10.1007/s11357-023-00738-0

**Published:** 2023-02-07

**Authors:** Jorge Almeida, Ana R. Martins, Lénia Amaral, Daniela Valério, Qasim Bukhari, Guilherme Schu, Joana Nogueira, Mónica Spínola, Ghazaleh Soleimani, Filipe Fernandes, Ana R. Silva, Felipe Fregni, Marcel Simis, Mário Simões, André Peres

**Affiliations:** 1 Proaction Lab, Faculdade de Psicologia e de Ciências da Educação, Universidade de Coimbra, Coimbra, Portugal; 2 CINEICC, Faculdade de Psicologia e de Ciências da Educação, Universidade de Coimbra, Coimbra, Portugal; 3 Psychological Assessment and Psychometrics Laboratory, Faculdade de Psicologia e de Ciências da Educação, Universidade de Coimbra, Coimbra, Portugal; 4 NOVA LINCS, University of Madeira, Caminho da Penteada, 9020-105 Funchal, Portugal; 5 Department of Biomedical Engineering, Amirkabir University of Technology (Tehran Polytechnic), Tehran, Iran; 6 Grupo HPA Saúde, Alvor, Portugal; 7 Spaulding Neuromodulation Center, Department of Physical Medicine & Rehabilitation, Spaulding Rehabilitation Hospital and Massachusetts General Hospital, Harvard Medical School, Boston, MA USA; 8 Faculdade de Medicina, Hospital das Clinicas HCFMUSP, Universidade de São Paulo, São Paulo, Brazil; 9 Department of Psychiatry, University of Minnesota, Minneapolis, USA; 10 Department of Neuroscience, Georgetown University Medical Center, Washington, USA

**Keywords:** Episodic memory decline, Cerebellum, Neurostimulation

## Abstract

Episodic memory decline is a major signature of both normal and pathological aging. Many neural regions have been implicated in the processes subserving both episodic memory and typical aging decline. Here, we demonstrate that the cerebellum is causally involved episodic memory under aging. We show that a 12-day neurostimulation program delivered to the right cerebellum led to improvements in episodic memory performance under healthy aging that long outlast the stimulation period — healthy elderly individuals show episodic memory improvement both immediately after the intervention program and in a 4-month follow-up. These results demonstrate the causal relevance of the cerebellum in processes associated with long-term episodic memory, potentially highlighting its role in regulating and maintaining cognitive processing. Moreover, they point to the importance of non-pharmacological interventions that prevent or diminish cognitive decline in healthy aging.

**Supplementary Information:**

The online version contains supplementary material available at 10.1007/s11357-023-00738-0.

## Introduction

As average life expectancy continues to steadily increase, by 2050, it is estimated that 1 in every 6 individuals in the world will be over the age of 65 [1]. This tectonic shift in demographics poses very significant challenges to individuals, families, and societies as a whole, because increased lifespan is strongly associated with cognitive decline, neurodegenerative disease, and overall frailty. Perhaps the major victim of age-related cognitive decline is episodic memory — our ability to recall past events seems to be severely affected in neurotypical and pathological aging [2–5]. Thus, understanding the relationship between the aging brain and episodic memory deficits, and developing interventions to ameliorate age-related episodic memory decline, is a major challenge for the neurosciences. Typically, the field has focused on the role (and age-related decline) of medial temporal lobe (MTL) structures, such as the hippocampus, and of the prefrontal cortex (PFC) in episodic memory [3, 4, 6]. However, a relatively recent emerging candidate for having a central role in episodic memory performance, and in overall cognitive decline under aging, is the cerebellum [7–13]. Here, we hypothesize that the cerebellum plays a causal role in episodic memory performance under healthy aging. To test this, we will use a randomized, double-blind, wait-list, and sham-controlled study to show that anodal transcranial direct current stimulation (tDCS) to the right cerebellum improves verbal episodic memory performance and enhances hippocampal functional and structural connectivity in healthy elderly individuals.

Episodic memory is one of the most affected processes under neurotypical and pathological age-related cognitive decline [3, 14, 15]. Episodic memory performance follows a steady and linear decline in normal aging beginning at the age of 50/60 years old [14, 15]. Concomitantly, structures known to be the backbone of episodic memory also show typical neural decline profiles with advanced aging. One such structure — the hippocampus [8, 16–19] — known to be central for episodic memory encoding, consolidation, and retrieval, is perhaps the single neural region that endures the most age-related decline. For instance, the hippocampus suffers severe volume loss, especially after the age of 50 years old [14, 16], along with reductions in synaptic plasticity and other neurobiological alterations [20]. Moreover, the hippocampus seems functionally decoupled from regions such as the posterior and anterior cingulate cortex under aging (and other default mode network — DMN — regions) [14, 21, 22]. Importantly, these neural changes correlate with age-related cognitive and episodic memory decline [20, 23]. In fact, the hippocampus is perhaps the first, and most severely affected, neural structure showing pathological change (e.g., volume loss) [24] under Alzheimer’s disease (AD) and its prodromal stage (mild cognitive impairment; MCI), and it is the best predictor of episodic memory impairment in AD [8, 24].

Another region that has been majorly implicated in episodic memory encoding and retrieval, particularly on those aspects that relate to cognitive control, rehearsal, and inhibition, is the prefrontal cortex [3, 4, 6, 8, 25]. Specifically, episodic memory-related activation in frontal and prefrontal lobes increases in the elderly, becoming more widespread and bilateral [14, 26]. That is, in the elderly, there is an attenuation of the activation asymmetry of the frontal and prefrontal lobes typical of young adults [14, 26]. This may function as a compensatory strategy [25] and, in fact, bilateral engagement of the PFC in episodic memory tasks is stronger in high-performing, when comparing to low-performing, older adults [25]. Moreover, there is marked decrease in white matter [27], and gray matter volume [2, 14, 27] around the PFC under aging.

A relative newcomer to the understanding of episodic memory and aging decline is the cerebellum. Traditionally, the cerebellum had been seen, exclusively, as the seat of motor coordination [28–30]. However, this has been heavily challenged in the last decades: the contribution of the cerebellum (and potentially those parts of the cerebellum that are more phylogenetically recent) goes well beyond that of motor coordination and impacts decisively on cognitive and emotional processes [7, 28–33]. For instance, the cerebellum has been shown to be involved among other things in verbal working memory [9, 11, 32], procedural learning [7], accurate timing of multiple signals and time judgments [7, 9], verbal learning [7], and language production [7]. Importantly, the cerebellum has also been associated with episodic memory performance (in particular verbal episodic memory) [7–13, 34, 35]. Episodic retrieval tasks lead to heightened activity in the posterior aspects of the cerebellum, along with hippocampal, prefrontal, anterior and posterior cingulate, and precuneus cortical activation [10]. Moreover, gray matter volume of these cerebellar regions has been shown to be correlated with episodic memory performance in delayed recall tasks [10, 34]. Finally, cerebellar lesions (especially within the right cerebellum) lead to, perhaps subtle, episodic memory deficits [11], in the context of other memory deficits related with working memory performance, suggesting that the integrity of the cerebellum is important for (episodic) memory function.

The role of the cerebellum in episodic memory is further demonstrated by cerebellar-cerebrum interactions, notably with the structures that have been associated with episodic memory — e.g., hippocampus. Interestingly, in both human and non-human animals, direct and indirect bidirectional projections between the hippocampus and the cerebellum have been shown [32, 35–39]. For instance, injections into hippocampi lead to staining of neurons within the cerebellum via the rhinal cortex, thalamus, and fastigial nucleus in several non-human animals (e.g., mice, cat) [32, 35–39]. Moreover, a direct white matter bundle connecting the hippocampus with the cerebellum (and namely Crus I and II among other lobes of the cerebellum) has been shown in humans [36, 39]. In line with this, diaschisis has been found after lesions in the cerebellar cortex, in that these lesions are associated with reduced cortical volume in medial temporal lobes [40]. Concomitantly, it has been suggested that the dentate gyri play a role in inhibiting overexcitation of the hippocampus [41]. Furthermore, aspects of the cerebellum seem to be functionally connected with, and implicated in, tasks that are supported by the hippocampus [32, 35, 39]. The connectivity between the cerebellum and the hippocampus may potentially support episodic memory functions that are related with cerebellar processing. In fact, synchrony between the hippocampus and the cerebellum at the theta band has been associated with memory encoding and retrieval performance [33], and enhancing this synchrony impacts episodic memory performance [33].

Importantly, the cerebellum has been shown also to be one of the most affected regions under normal aging. For instance, the cerebellum shows reductions in gray matter volume and white matter integrity as a function of aging to the same level as those observed in PFC and only slightly less severe than those observed within the hippocampus [9, 14, 31, 34]. Moreover, the cerebellar volume correlates with scores in memory tests [9, 13, 31, 42], and the cerebellum shows under activation in memory and other cognitive tasks under aging [34]. In line with this volumetric reduction and functional under activation, the connectivity of the cerebellum is also affected by age-related neural decline [34]. Specifically, its connectivity with the MTL and DMN regions has been shown to be severely affected in the elderly [31, 34, 37]. Perhaps not surprisingly, the cerebellum is now clearly linked also to pathological aging. For instance, amyloid plaques have been shown in the cerebellum of a large number of AD patients but not of control individuals [43]. Furthermore, cerebellar atrophy is not only present in AD, but correlates with scores in dementia scales [34]. Likewise, cerebellar atrophy correlates with episodic memory scores in MCI patients [13]. Finally, functional connectivity between the cerebellum and the hippocampus is also affected in MCI patients, impacting their episodic memory retrieval abilities [8, 31, 37].

**Fig. 1:**
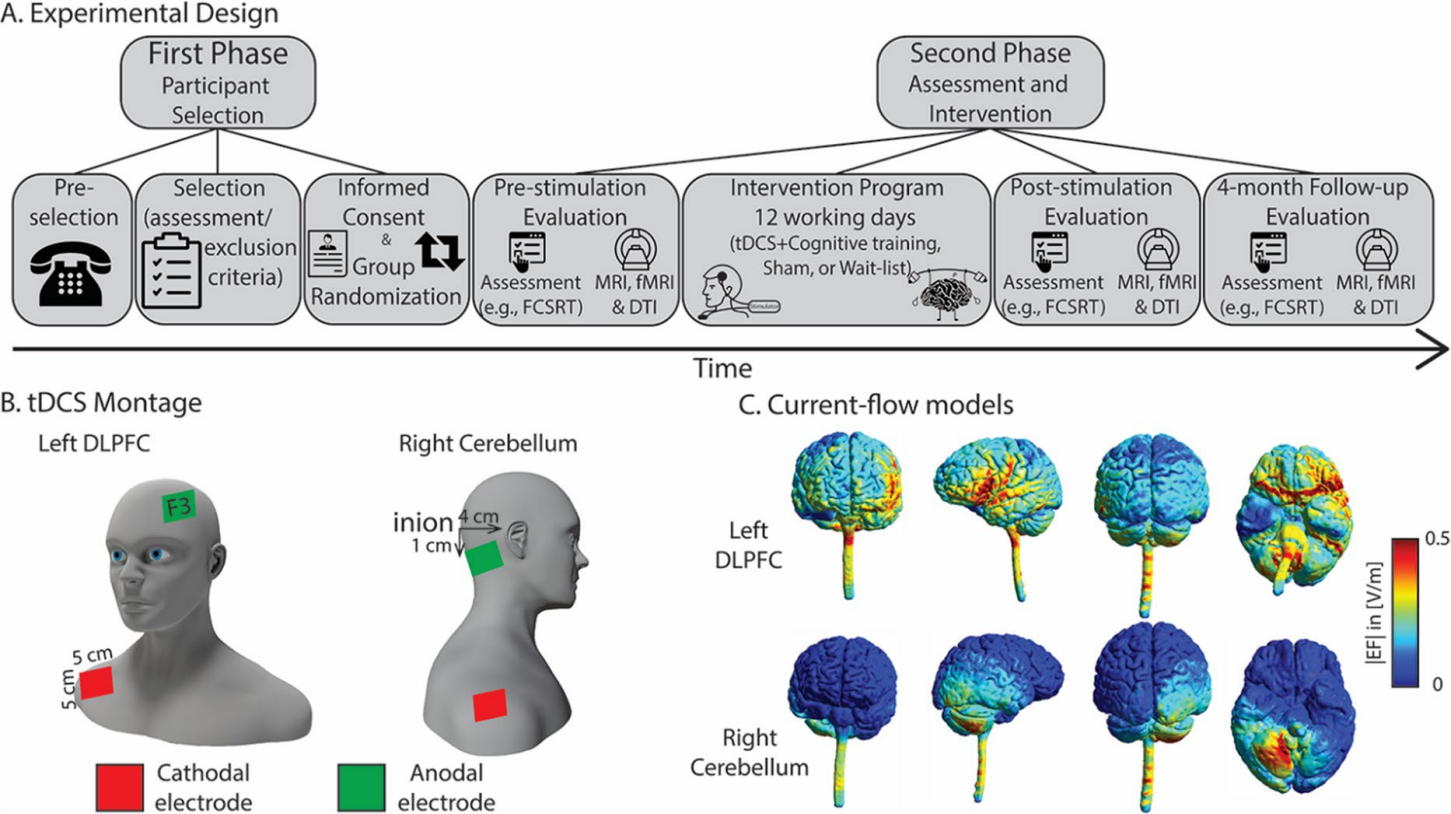
Methods and tDCS montage. In (A), we show a schematic of the implementation of our experimental design, from participant selection to assessment and intervention; in (B), we show schematics of the two different tDCS montages; in (C), we show the electric field strength of a randomly selected healthy subject for two electrode montages when a current of 2 mA was used. Simulations were run on SimNIBS 3.2

Based on the state-of-the-art presented above, we propose that the cerebellum is a major structure in age-related cognitive decline, and specifically episodic memory decline. Moreover, we predict that the role of the cerebellum in episodic memory relates mechanistically to how the cerebellum interacts with the connectivity fingerprints of the hippocampus that are at play in the service of episodic memory. We will test these hypotheses causally. Specifically, we devised a multi-session (12 consecutive working days), randomized, double-blind, wait-list and sham-controlled, multi-site (right cerebellum or left DLPFC) anodal tDCS stimulation coupled with cognitive training study in healthy elderly individuals. We collected different dependent variables at three different time points: at a pre-stimulation time (a day before the start of the program); at a post-stimulation time (1 day after the stimulation program); and at a 4-month follow-up visit. As a primary dependent variable, we collected delayed recall performance with the *free and cued selective reminding test* (FCSRT) [44, 45] — the FCSRT is one of the most used standardized tests to assess episodic memory performance, and includes normative data for aging populations in Portugal [44, 45]. As secondary dependent variables, we collected functional magnetic resonance imaging (fMRI) resting scans and diffusion tensor imaging (DTI) data. As a primary outcome, we tested episodic recall differences between the pre- and post-stimulation evaluation times and between the pre-stimulation and 4-month follow-up evaluation times for each experimental group. As a secondary outcome, we tested hippocampal functional connectivity changes, and diffusion changes in typical memory-related white matter tracts after tDCS stimulation (immediate and at the 4-month follow-up evaluation). Participants were pseudorandomly assigned to one of four experimental groups: a wait-list group — where participants were not subjected to any stimulation protocols between the pre- and post-stimulation sessions; a sham control group — where participants went through tDCS montage but stimulation was applied only within the first 30 s; a left DLPFC group — where anodal tDCS was applied to the left DLPFC; and finally, a cerebellum group — where anodal tDCS was applied to the right cerebellum. Stimulation was applied to the right cerebellum because the episodic memory effects presented above seem to be stronger for the right cerebellar hemisphere (e.g., [9, 31, 46]). For all the groups except for the wait-list group, on each one of the 12 days, participants would first go through tDCS (real or sham) stimulation for 20 min. They would then go through a 1-h cognitive training program where they would perform a series of paper and pencil and computerized games and tasks related with memory (see Fig. 1). We show that tDCS to the right cerebellum leads to immediate and long-term improvement of verbal episodic memory performance and enhances hippocampal functional and structural connectivity, demonstrating the causal role of the cerebellum in episodic memory.

## Materials and methods

### Participants

Fifty-six healthy elderly individuals (≥ 60 years old) were included in the study (34 female, 22 male; mean age = 68.5; mean formal education years = 12.3; see Supplementary Table 1 for demographic information). Participants were recruited through broad-based advertisements in the community (e.g., flyers, websites, public talks) and referral institutions (e.g., senior universities, community health centers, nursing homes). All the participants were Portuguese native speakers, were right-handed (as assessed by the Laterality tasks of the “Coimbra Neuropsychological Assessment Battery) [47], had no history of neuropsychiatric disorders (e.g., stroke, epilepsy, dementia, depression) or head injury, had no metallic implants, did not intake concurrent medication likely to affect cognition, and had no history of alcohol and drug abuse. Written informed consent was obtained from all participants prior to the beginning of the study. Participants were each paid €50 upon completion of the study. This study was approved by the Ethics Committee of the Faculty of Psychology and Educational Sciences of the University of Coimbra, and performed following the ethical principles of research with human subjects.

### Procedure

Pre-selection eligibility assessment was performed by phone and in-person before the study. Here, we checked for major inclusion and exclusion criteria (those mentioned above; e.g., medication, metal implants, laterality). Afterwards, participants went through a screening session. In this session, we assessed global cognitive function, using the Montreal Cognitive Assessment (MoCA) [48–50], and functional abilities using the Adults and Older Adults Functional Assessment Inventory [51, 52]. All participants performed above the cutoff for mild cognitive impairment, according to their age and educational level [49], and all except two showed functional disability scores in activities of daily living between 0 and 10%. Two participants revealed higher percentages of global functional disability (> 14%), but these cognitive difficulties were not confirmed by MoCA scores and their physical limitations did not hamper their participation in the study. Finally, we also measured depression using the Geriatric Depression Scale (GDS) [53–55]. None of the participants showed severe depressive symptomatology.

Upon inclusion in the study, participants were randomly assigned (1:1:1:1) to the following groups: (1) anodal tDCS to the left DLPFC plus cognitive training; (2) anodal tDCS to the right cerebellum plus cognitive training; (3) sham tDCS plus cognitive training; (4) wait-list group. Participants allocated to the first three conditions were not informed as to what group they were allocated, neither whether they were receiving either active or sham tDCS stimulation. A minimal sample size of 11 participants per experimental group was determined taking into consideration that the primary outcome would be analyzed using a repeated measures ANOVA in a four-arm study with a significant level *α* of 5% with Bonferroni’s adjustment for multiple comparisons, an 80% power for a detection of a 1.5 increase in the *z*-score value of verbal episodic memory with a standard deviation of 1 [56]. Considering potential dropouts, the total number of participants per group in this study was set at 14 (56 in total). All participants completed 15 study sessions: the baseline assessment, 12 stimulation sessions, the post-intervention assessment, and a 4-month follow-up. The 12 stimulation sessions were conducted on consecutive weekdays, at the same time of the day, and comprised 20 min of anodal tDCS (either to the left DLPFC or to the right cerebellum), followed by 1 h of computer-based and pen-and-paper cognitive training tasks. Both the participant and the researcher administering cognitive training were blind to the experimental condition and only the researcher applying tDCS was aware of the allocation of participants per group. The assessment sessions (baseline, post-intervention, and follow-up) included a neuropsychological assessment protocol, carried out by an external blinded rater, and a MRI, DTI. and rest-fMRI session. After completion of the intervention sessions (session 12), participants in the tDCS training conditions were asked to guess whether they had received active or sham tDCS. Participants were unblinded to their experiment condition after the follow-up session.

### Cognitive training tasks

The cognitive training program consisted of 12 sessions of computerized and pen-and-paper exercises focused on memory training, specifically verbal episodic memory. The training was organized in 6 sessions per modality (computer/pen-and-paper), administered in alternate order to increase the interest of the participants throughout the session. For the interactive computerized exercises, we used memory training tasks from the RehaCom cognitive rehabilitation software (Hasomed Inc, Magdeburg, Germany) that have been shown to enhance memory in healthy aging and mild cognitive impairment (namely its memory training modules; e.g., Memory for Words module and Physiognomic Memory) [57, 58]. Pen-and-paper tasks focused also on memory training exercises that included a working memory task, a semantic memory task, a face memory task, and an autobiographical memory task taken from a Portuguese paper and pencil memory training program (Memo +) [59].

Before each exercise, participants were first given a verbal explanation of the tasks to be performed. In the computerized tasks, all participants began training at the beginner’s level of the RehaCom software. The training modules automatically adapted the training tasks to the user’s level of performance — according to whether the participant succeeded or failed the task, the difficulty levels were automatically adjusted to meet the participant’s ability. The same procedure was applied to the pen-and-paper tasks, where the difficulty levels were always adapted to each participant’s performance. All participants were trained on the same tasks for the same time, and the duration of each session was 1 h (plus 20 min of tDCS).

### tDCS montage

Anodal tDCS was applied daily for 12 consecutive weekdays in all experimental sessions using a TCT Stimulator Model 101 (Research Limited, Hong Kong, China). From the four intervention groups mentioned above, two received active tDCS and one received sham tDCS. In the active tDCS groups, electric stimulation was given continuously for 20 min at an intensity of 2 mA. The anode electrode was either placed on the left DLPFC, over the F3 location according to the 10–20 EEG international system, or over the right cerebellar cortex, following the setup proposed by Pope and Mial [60] — 1 cm under and 4 cm lateral to the inion. In the sham condition, current was applied for 60 s only (30 s ram up and 30 s down), and the electrodes were placed as in the DLPFC montage. It should be noted that less than 3 min of tDCS induces no effects on cortical excitability [61] and using 60 s of stimulation is a reliable method of blinding, as it induces similar sensations on the scalp as real tDCS. For each montage, the reference electrode (cathode) was applied over the right deltoid muscle. The extra-cephalic reference was used to avoid the possible confounding effects that may be induced by two electrodes with opposite polarities. For all conditions, the anode and cathode rubber electrodes (5 × 5 cm, 25cm^2^) were placed inside a sponge soaked in saline solution and held in place by cloth straps with Velcro (see Fig. 1B and C for tDCS montage and current flow maps). Throughout the duration of each session, the participants were accompanied by a researcher, who continuously monitored the current intensity and impedance. The tDCS device was placed by the researcher, while s/he was behind the participant, to preserve blinding. After every tDCS session, the stimulation sites were checked for side effects and a questionnaire was filled out.

### Neuropsychological assessment

The neuropsychological assessment sessions were conducted before, immediately after, and 4 months after the intervention to assess its long-term effects. Episodic memory performance was the primary outcome of this study. To assess episodic memory performance, we used the Portuguese version of the Free and Cued Selective Reminding Test (FCRST) [44, 45]. FCRST is an instrument that assesses verbal memory and learning through the presentation of a 16-word list memory test. There are three rehearsals of free and cued reminding (with 20 s of an interfering exercise between them) and a delay recall test (30 min after). Our outcome variable was exclusively the unguided delay recall test. A parallel version of FCRST was used in the post-intervention assessment session, in order to avoid learning and practice effects. The parallel version differed exclusively on the words presented, maintaining the number of words and respective categories.

Neuropsychological assessment included a series of other neuropsychological tests that were not considered primary outcomes and are not analyzed in the present study. These included the following instruments: (1) Subjective Memory Complaints (SMC) [62, 63], used to characterize memory complaints; (2) Continuous Visual Memory Test (CVMT) [64, 65], which evaluates visual memory ability; (3) Toulouse-Piéron Cancellation Test [66, 67], a cancellation task that evaluates selective and sustained attention; (4) “Symbol Search” and “Digit Symbol-Coding” — subtests of the Wechsler Adult Intelligence Scale (WAIS-III) [68, 69], which measure information processing speed, visual perception, speed of processing, and executive functioning; (5) Stroop [70], which assesses the ability to inhibit interference; (6) Semantic Verbal Fluency Test (SVF) [71–73], which evaluates the abilities of processing speed, language production, and executive functions; and (7) World Health Organization Quality of Life-Older Adults Module (WHOQOL-OLD) [74–76], which assesses self-perceived quality of life. Average scores per group on these tests are presented in supplementary table 2.

### MRI acquisition and preprocessing

Whole-brain fMRI data were collected with a 3-T Siemens MAGNETOM trio MRI scanner (Siemens Healthineers, Erlangen, Germany) using a standard 12-channel head coil. High-resolution structural MRI data were acquired using a T1-weighted magnetization prepared rapid gradient echo (MPRAGE) sequence, which entailed the following parameters: 256 × 256 acquisition matrix, 256 mm field-of-view (FoV), voxel size of 1.0 × 1.0 × 1.0 mm^3^, flip angle (*α*) of 7°, bandwidth (BW) of 200 Hz/px, repetition time (TR) of 2530 ms, and an echo time (TE) of 3.29 ms. For resting-state fMRI data acquisition, participants were instructed to remain awake, laid supine, and with their eyes open, looking to a fixation cross, for 6 min. We used a T2*-weighted gradient echo-planar imaging (EPI) sequence with the following specifications: 64 × 64 acquisition matrix, 256 mm FoV, flip angle of 90°, 33 interleaved slices, voxel size of 4.0 × 4.0 × 4.0 mm^3^, BW of 1562 Hz/px, TR of 2200 ms, and TE of 30 ms. Finally, diffusion-weighted images (DWI) were acquired using a single-shot echo-planar sequence, with 79 diffusion directions, TR of 8900 ms and TE of 86 ms, diffusion weighting factor (*b*) of 1000 s/mm^2^, 70 slices with isotropic voxel resolution of 2 × 2 × 2 mm^3^, and 10 non-diffusion-weighted (*b* = 0 s/mm^2^) volumes.

Both anatomical and functional data were preprocessed using fMRIPrep 20.1.1. This pipeline includes standard preprocessing steps, and its implementation aims to respond to the lack of an easy-usage workflow that ensures robustness independently of the data idiosyncrasies, and to increase consistency of fMRI results [77]. For each of the 3 resting-state runs obtained per subject (one for each session: pre-stimulation evaluation time, post-stimulation evaluation time, and follow-up), the following preprocessing was performed. First, the five first volumes were skipped and a reference BOLD volume and its skull-stripped version were generated. Head-motion parameters with respect to the BOLD reference (transformation matrices, and six corresponding rotation and translation parameters) were estimated before any spatiotemporal filtering was employed using mcflirt (FSL 5.0.9) [78]. BOLD runs were slice-time corrected using 3dTshift from AFNI 20160207 [79] (RRID: SCR_005927). The BOLD reference was then co-registered to the T1-weighted image reference using bbregister from FreeSurfer (version 6.0.0) [77] which implements boundary-based registration [80]. The BOLD time series were resampled onto the MNI152NLin2009cAsym using the combined volumetric and surface-based (CVS) registration from Freesurfer (version 6.0.0) [81] and smoothed using SUSAN [82] with brightness threshold equal to 75% of the median brightness of the input image, FWHM of 6 mm, and the Univalue Segment Assimilating Nucleus (USAN) was defined over the reference BOLD volume. Non-aggressive AROMA denoising (ICA-AROMA) [83] was performed on the registered functional data using the FilterRegressor function from Nipype (version 1.3.2) [84]. Additionally, we calculate the CSF and WM average temporal series after AROMA denoising (NiftiLabelsMasker—Nilearn version 0.6.2) [85] and regressed them out, alongside the six head-motion parameters using the TProject from Nipype (version 1.3.2) [84]. Finally, we performed a second-order polynomial detrending and a temporal bandpass filtering of 0.009 and 9999 Hz.

Diffusion data was preprocessed using an in-house pipeline written in MATLAB R2019a that combined features of the FDT diffusion module from the FMRIB (Functional Magnetic Resonance Imaging of the Brain’s diffusion toolbox) Software Library (FSL, version 6.0.4), and tools from the MRtrix3 software (https://www.mrtrix.org/; the codes are available at https://github.com/maismemoria/preprocessing). First, the acquired DWI data were converted from DICOM to NIFTI and inspected for visual quality. For each subject, images were denoised using the MP-PCA method [86] and non-weighted diffusion images (b0s) were used to generate a whole brain mask [87]. Next, participant motion and eddy current distortions were corrected using the *eddy_openmp* program, which is part of the FSL FDT toolbox [88]. Diffusion tensor was estimated via least square fitting and the mode of anisotropy (MA) measure was computed to characterize the diffusion displacement in the microstructure of white matter. The MA measure [89] is a real value defined in the interval between − 1 and 1, and indicates how planar or linear is the tensor, respectively. A planar diffusion tensor is characterized to have two large eigenvalues and one small eigenvalue, whereas a linear diffusion tensor is characterized to have one large eigenvalue and two small eigenvalues. Studies have reported that the MA can detect white matter alterations that other diffusion measures cannot [90].

### Data analysis

For our primary outcome (i.e., episodic memory performance), a 4 (experimental condition: wait-list; sham; left DLPFC; right cerebellum) × 3 (assessment times: pre-stimulation evaluation time; post-stimulation evaluation time; 4-month follow-up) repeated measures ANOVA was calculated over the episodic memory performance (i.e., the delayed FCSRT measure) of our participants. Our main interest was the experimental condition * assessment time interaction, and specifically the simple effects comparing for each group the pre- versus the post-stimulation evaluation time and the pre-stimulation evaluation time versus the 4-month follow-up. We used Bonferroni-corrected post hoc *t*-tests to test for these comparisons.

In what regards our secondary outcomes (i.e., the MRI functional and structural connectivity), we used different analytical pipelines. For the functional connectivity analysis, we used two approaches: a region-wise strategy, where we defined regions-of-interest (ROI) and calculated functional connectivity between the hippocampi and a set of predefined ROIs, and a whole brain voxelwise approach where we calculated functional connectivity of the hippocampi with every voxel in the brain.

For the ROI analysis, we defined the ROIs to be used based on a Neurosynth meta-analysis map provided by the uniformity test (https://neurosynth.org/) using the keywords “episodic memory” (332 studies were included, FDR corrected < 0.01). The Episodic Memory Neurosynth map was resampled onto the MNI152NLin2009cAsym (isovoxel of 4 mm) using the combined volumetric and surface-based (CVS) registration from Freesurfer (version 6.0.0) [91]. Then, we applied a simple cluster algorithm (first-neighbors contiguous voxels) in order to segregate the Neurosynth map in discrete regions. In each of the output clusters, we identified the voxel with the highest value, and using it as a seed of a region-growing algorithm, we used the highest 50 contiguous voxels (3.2 cm^3^) to fill in the ROI around the peak value. In total, we found 19 ROIs that, at least in part, overlap with the following anatomic regions: left hippocampus, right hippocampus, anterior cingulate cortex (ACC), ventromedial prefrontal cortex (PFC), left inferior frontal gyrus, right inferior frontal gyrus, left anterior insula, right anterior insula, left parahippocampal gyrus, right parahippocampal gyrus, dorsal posterior cingulate cortex (PCC), left ventral PCC, right ventral PCC, left precuneus, right precuneus, left intraparietal sulcus, right intraparietal sulcus, left angular gyrus, and right angular gyrus. After selecting the ROIs, for each of the three sessions, we calculate the functional connectivity (Pearson correlation Fisher transformed) of the left and right hippocampus with all other selected regions producing a connectome with 35 unique pairs. We then tested which ROI pairs of the post-stimulation and follow-up connectomes showed higher co-activation than in the pre-stimulation connectome, using a paired one-tail two-sample *t*-test. The results were corrected by the false discovery rate independently for each condition (35 simultaneous statistical inferences; FDR < 0.05) [92].

For the whole brain voxelwise analysis, we used the same left and right hippocampus ROIs defined in the ROI-to-ROI analysis as the seeds to calculate the seed-to-voxel connectivity. The BOLD volumes were gray matter masked, ensuring that all non-zero voxels of all subjects’ gray matter masks (automatically defined by fMRIPrep) were included. For each seed, we applied a general linear model using its average time series as the design matrix, resulting in 6 beta maps (two seeds and three sessions) per subject. Next, we compared whether there were differences in the beta maps of the post-stimulation and follow-up evaluation times compared to the pre-stimulation evaluation time. To test these hypotheses, we used the function Randomise two-sample paired *t*-test with 5000 permutations and corrected by threshold-free cluster enhancement (TFCE; FSL package) [93, 94] and for multiple comparisons (*p*FWE-corrected < 0.05).

In the structural connectivity analysis, we examined three white matter tracts that have been previously reported to be associated with episodic memory, the fornix, the uncinate fasciculus, and the cingulum bundle [95–98]. The fornix is known to be associated with episodic memory performance in various neuropathological conditions [95]. Similarly, degradations in the uncinate fasciculus have been reported to affect memory components [99]. Lastly, it has been suggested that alterations in the cingulum bundle precede hippocampal atrophy influencing episodic memory performance in different syndromal stages [100, 101]. We estimated these tracts using the Tract-Based Spatial Statistics package (TBSS) [102]. Briefly, the computed diffusion tensor maps were non linearly registered, aligning each subject and each measure map into a common space (FMRIB58_FA — 1 × 1 × 1 mm^3^). For each measure, we created average maps and their corresponding skeletons (thinned measured maps containing the centers of all white matter tracts common to a group of interest) [102]. Then, voxelwise statistical analysis, constrained by the ROIs in the white matter skeleton (JHU-DTI atlas) [103], was computed using Randomise paired *t*-test with 5000 permutations using TFCE [94]. Results were corrected for multiple comparisons independently for each white matter skeleton (*p*FWE-corrected < 0.05). Most analyses were performed at the LCA cluster at the University of Coimbra.

## Results

### Is the cerebellum causally involved in episodic memory?

Delayed free recall measures from the FCSRT (henceforth episodic memory performance) at the three different data collections moments per stimulation group are presented in Table 1. First, we analyzed episodic memory performance for each group at the pre-stimulation time, and demonstrated that there were no statistically significant group differences in the ability to free recall the items in the FCSRT prior to the stimulation program (*F* (3,52) < 1; partial *η*^2^ = 0.05) — that is, our experimental groups do not differ in their baseline episodic memory performance. Next, we examined the effect of group membership and stimulation protocols on episodic memory performance as a function of time (i.e., the pre-, post-, and follow-up data collection times). There was a significant increase in episodic memory performance with time (*F* (2,100) = 23.929; *p* < 0.001; partial *η*^2^ = 0.324). That is, participants were better at recalling items as a function of the time of data collection. This may be due to training effects on the FCSRT. Specifically, we used two different versions of the list of words for participants to memorize: in the pre-stimulation and follow-up conditions, the same list of words was used, whereas for the post-stimulation condition, a different list of words was used. Thus, both task and list training effects may be present in the follow-up condition, whereas only task training effects may be present in the post-stimulation condition. Moreover, there were no significant differences in episodic memory performance between the groups (*F* (3,50) = 2.266; *p* = 0.092; partial *η*^2^ = 0.12).

**Table 1:** Episodic memory performance by evaluation time and experimental group

		Time		
		Pre-stimulation	Post-stimulation	Follow-up
Experimental group	Wait-list	10.57 (0.83)	10.43 (0.77)	11.38 (1.06)
	Sham	9.43 (1.03)	11.14 (0.88)	11.31 (1.06)
	Left DLPFC	11.07 (0.57)	12.93 (0.46)	12.71 (0.74)
	Right cerebellum	10.86 (0.51)	13.21 (0.48)	13.86 (0.39)

Average episodic memory performance and SEM values

**Fig. 2:**
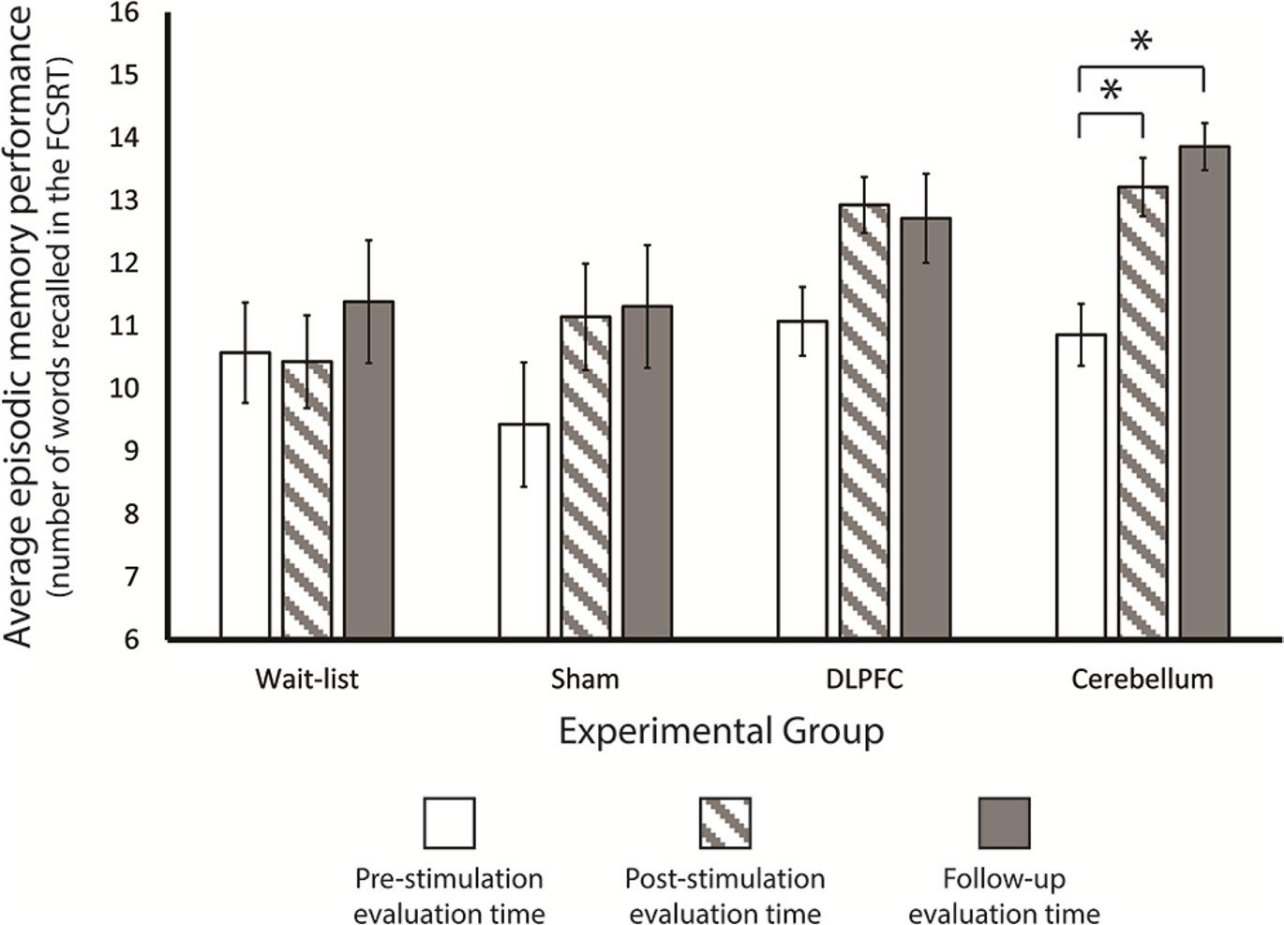
Behavioral results. Here, we show the number of recalled words in the delayed free recall task of the FCSRT per experimental group and per evaluation time. The only significant differences between the pre-stimulation evaluation time and the remaining evaluation times were observed for the right cerebellum anodal tDCS group — i.e., the cerebellum group showed an improvement in episodic memory performance in both post-stimulation evaluation times when compared to the pre-stimulation evaluation time. Error bars correspond to the standard error of the mean; *p-values below 0.05 Bonferroni corrected; error bars correspond to SEM

Importantly, a significant interaction between data collection time and group membership was obtained (*F* (6,100) = 2.546; *p* = 0.025; partial *η*^2^ = 0.133). Post hoc *t*-tests on the simple effects were performed between pre- and post-stimulation and pre-stimulation and follow-up evaluation times per experimental group (Bonferroni critical *p*-value 0.00625; see Fig. 2). tDCS stimulation to the right cerebellum led to a statistically significant increase in episodic memory performance from the pre- to the post-stimulation evaluation (*t* (13) = 5.518; *p* < 0.0001). For all the other experimental groups, episodic memory performance between pre- and post-stimulation was not significantly different (wait-list: *t* (13) < 1; sham: *t* (13) = 2.747; *p* = 0.017; DLPFC: *t* (13) = 2.959; *p* = 0.011). A similar effect was present when we compared episodic memory performance in the pre-stimulation evaluation and in the follow-up evaluation: cerebellum anodal tDCS stimulation led to a significant improvement in episodic memory performance in the follow-up evaluation when compared with the performance at the pre-stimulation evaluation (*t* (13) = 10.817; *p* < 0.0000001), whereas the other stimulation conditions did not lead to statistically significant differences in memory performance (wait-list: *t* (12) = 1.011, *p* = 0.332; sham: *t* (12) = 2.759; *p* = 0.017; DLPFC: *t* (13) = 2.703; *p* = 0.018).

If we look more leniently to our results (i.e., no correction applied), individuals in both the sham and DLPFC groups show some memory improvement both at the post-stimulation and follow-up evaluation when compared to the pre-stimulation evaluation, and this effect may be perhaps stronger for the DLPFC group. This effect may be related with the importance of frontal cortices in episodic memory performance. However, it may also be related with a general effect cognitive training in episodic memory performance, or the overall list and task training due to test (but see the performance of the wait-list group) and list repetition.

Importantly, these primary outcome results demonstrate that after right cerebellum anodal tDCS stimulation, healthy elderly individuals show very clear episodic memory improvement — on average of about 3 items (out of 16) — both 1 day after the 12-working day stimulation program and 4 months after this program, causally demonstrating that the cerebellum is involved in episodic memory performance. Notably, 11 out of the 14 participants in the cerebellum group showed an improvement in episodic memory performance at the post- compared to the pre-stimulation evaluation times (with the remaining 3 showing no difference between the two evaluation times), whereas all of the 14 participants showed improvement in episodic memory performance at the follow-up when compared to the pre-stimulation evaluation time.

### Does tDCS stimulation to the cerebellum affect functional connectivity of the hippocampus?

**Fig. 3:**
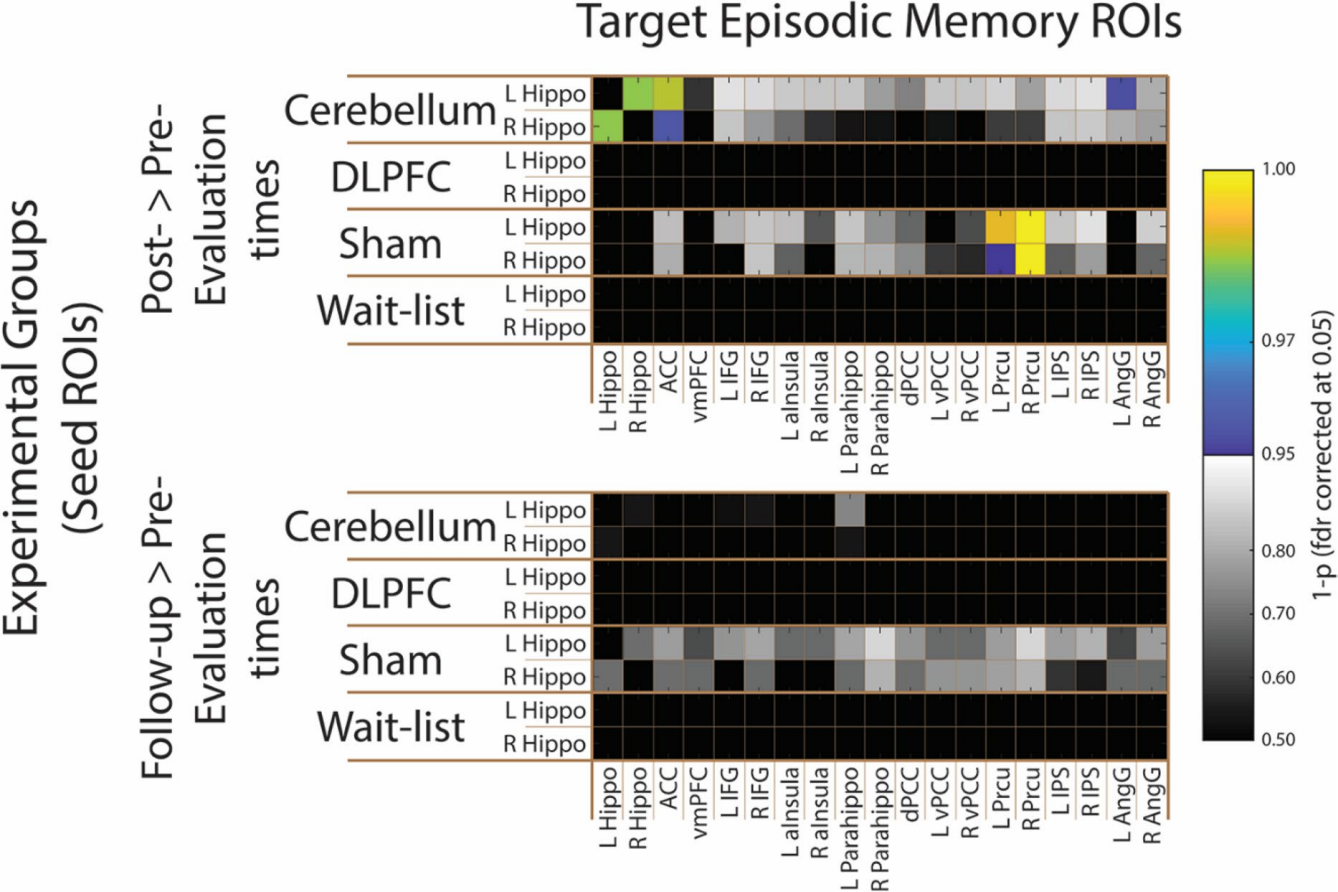
ROI-to-ROI neural effects of tDCS stimulation. Here, we show ROI pairwise differences in functional connectivity values per group and per temporal comparison (pre- versus post-stimulation evaluation times, and pre-stimulation versus follow-up evaluation times) using as seed ROIs the left and right hippocampus, and as target ROIs areas that are known to be integral part of the episodic memory network. Specifically, the connectivity between the left and the right hippocampus, and between these and the ACC and left angular gyrus was higher in the post- than in the pre-stimulation evaluation times in the cerebellum experimental group. Moreover, the connectivity between the hippocampi and the precuneus was also heightened in the sham experimental group. No significant effect was observed in the comparison between the pre-stimulation evaluation time and the follow-up. *p*-values are FDR corrected. The blue-yellow color map indicates the pairs of ROIs that obtained 1 minus *p*-value (FDR corrected for 35 comparisons) higher than 0.95, while the grayscale color map represents the pairs with 1 minus *p*-values lower than 0.95. L Hippo, left hippocampus; R Hippo, right hippocampus; ACC, anterior cingulate cortex; vmPFC, ventromedial prefrontal cortex; L IFG, left inferior frontal gyrus; R IFG, right inferior frontal gyrus; L aInsula, left anterior insula; R aInsula, right anterior insula; L Parahippo, left parahippocampal gyrus; R Parahippo, right parahippocampal gyrus; dPCC, dorsal posterior cingulate cortex; L vPCC, left ventral posterior cingulate cortex; R vPCC, right ventral posterior cingulate cortex; L Prcu, left precuneus; R Prcu, right precuneus; L IPS, left intraparietal sulcus; R IPS, right intraparietal sulcus; L AngG, left angular gyrus; R AngG, right angular gyrus

tDCS stimulation to the right cerebellum consistently improved episodic memory performance, but did it also consistently affect the connectivity fingerprints of the hippocampus — as the major area involved in episodic memory processing? To address this question, we focused first on whether our experimental conditions changed how the hippocampus communicates with other regions that are also typically implicated in episodic memory. We computed the difference in functional connectivity scores between the left and the right hippocampus with a series of regions that are also involved in episodic memory (see methods for the selection of the particular regions-of-interest; ROIs) for the pre- versus the post-stimulation evaluation times, and for the pre-stimulation versus the follow-up evaluation times. We found significant differences in the functional connectivity fingerprints of the hippocampus for the post-stimulation condition, when compared to the pre-stimulation condition, for the cerebellum group. Specifically, the connectivity of the left hippocampus with the left angular gyrus and the anterior cingulate cortex (ACC), in the vicinity of Brodmann Area 8, was stronger for the post-stimulation evaluation time, when compared to pre-stimulation time (left angular gyrus: *t* (13) = 2.98, *p* FDR corrected = 0.046; ACC: *t* (13) = 4.36, *p* FDR corrected = 0.014). Moreover, the right hippocampus also showed heightened functional connectivity with the ACC for the post-stimulation evaluation time, when compared to the pre-stimulation time in the cerebellum group (*t* (13) = 3.14, *p* FDR corrected = 0.045). Finally, connectivity between the hippocampi was also heightened immediately after the intervention for the cerebellum group (ACC: *t* (13) = 3.91, *p* FDR corrected = 0.016; see Fig. 3), suggesting stronger cross talk between these three regions. No significant effects were found when we compared the pre-stimulation and the follow-up evaluation times for the cerebellum group.

We also found changes in the profile of functional connectivity of the hippocampi for the sham group when comparing the pre-stimulation with the post-stimulation evaluation times. Specifically, both hippocampi show heightened connectivity with the precuneus bilaterally (left hippocampus-left precuneus: *t* (12) = 4.00, *p* FDR corrected = 0.010; left hippocampus-right precuneus: *t* (12) = 5.69, *p* FDR corrected = 0.002; right hippocampus-left precuneus: *t* (12) = 2.98, *p* FDR corrected = 0.050; right hippocampus-right precuneus: *t* (12) = 5.23, *p* FDR corrected = 0.002; see Fig. 3). No other comparisons for the sham group or the other experimental groups were statistically significant.

These data show that tDCS to the cerebellum changes how the hippocampus communicates with the remaining regions implicated in episodic memory — i.e., the effects of stimulating the cerebellum on episodic memory performance are accompanied by heightened connectivity between the hippocampus and the left angular gyrus and the ACC — two structures that play a role in episodic memory performance, and particularly in retrieval of information [5, 104–106], and are part of the DMN [107] — as well as higher levels of connectivity between the hippocampi. Interestingly, the sham group presented heightened connectivity between the hippocampus and the precuneus — an area that has been implicated in episodic memory and the DMN [106–108], but has also been shown to be associated with cognitive training enhancements [109]. All these results were, however, obtained only for the comparison between the pre- and the post-stimulation conditions.

Secondly, we then looked at whether there were more widespread effects of tDCS stimulation plus cognitive training intervention on the functional fingerprints of the hippocampus.

**Fig. 4:**
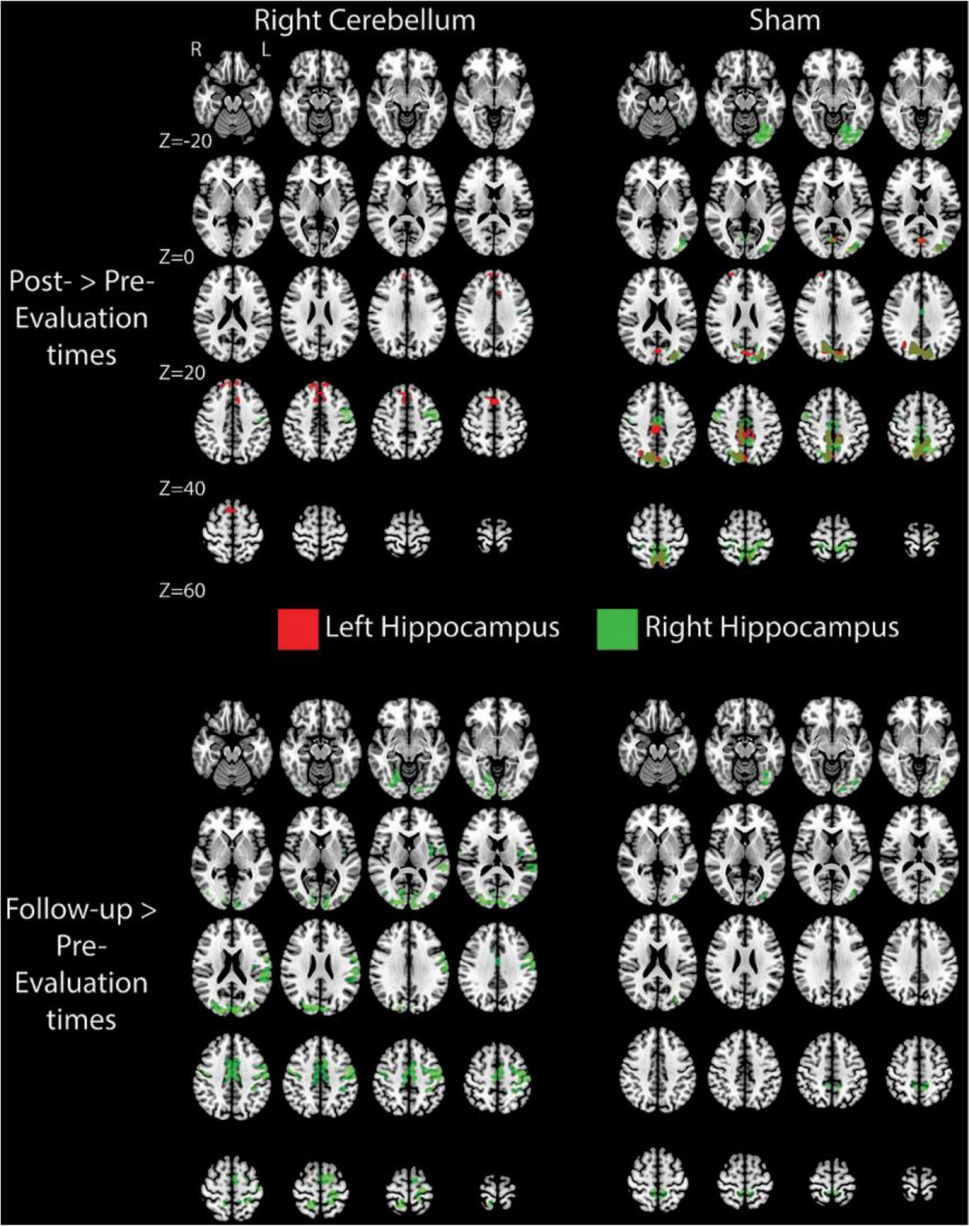
Whole-brain neural effects of tDCS stimulation. Here, we show significant whole-brain differences in functional connectivity values per group and per temporal comparison (pre- versus post-stimulation evaluation times, and pre-stimulation versus follow-up evaluation times) using as seed ROIs the left and right hippocampus. We show widespread functional connectivity enhancements in the follow-up comparison (and the less widespread for the post-stimulation comparison) for the cerebellum experimental group, specifically in regions that have been shown to be important for episodic memory and that are part of the DMN. Less widespread effects were observed also for the sham group (and are more restricted to the precuneus and occipitotemporal regions). No other effects (for all time comparisons and experimental groups) were significant. All maps are thresholded at *p* < 0.05 FEW corrected

Specifically, we computed whole brain connectivity maps for each hippocampus, by experimental group and evaluation time. We then compared, for each experimental group independently, whether whole-brain hippocampus connectivity was affected by the intervention between the pre- versus the post-stimulation evaluation times and between the pre-stimulation versus follow-up evaluation times. Overall, stimulating the right cerebellum led to relative widespread changes in the connectivity of the hippocampus (all maps *p*FWE-corrected at 0.05; see Fig. 4). Specifically, when we compared the pre- to the post-stimulation evaluation times, the right hippocampus showed heightened connectivity with a large cortical swath in the left superior frontal cortex. Importantly, these regions have been associated with retrieval of episodic details and rich contextual associations [106, 108, 110], as well as with monitoring and selecting important information (and inhibiting unimportant information) for retrieval [106, 111]. Moreover, the left hippocampus showed heightened connectivity with the PFC — namely its dorsal and medial aspects — bilaterally extending to the dorsal ACC. This heightened connectivity may relate to the role that the dorsal and medial aspects of the PFC have in deep episodic encoding of verbal material [12, 104, 112]. Furthermore, connectivity of the left fusiform gyrus and lingual gyrus to the left hippocampus also showed to be heightened right after tDCS stimulation to the right cerebellum. Interestingly, the left fusiform and lingual gyrus have been associated with successful shallow episodic encoding and spatial memory [104, 113] (see Fig. 4).

When we looked at more stable and long-lasting effects of tDCS stimulation over the right cerebellum on the connectivity profiles of the hippocampi (i.e., when we compared the pre-stimulation with the follow-up evaluation times), we observed extensive changes in the connectivity fingerprints of the right hippocampus (see Fig. 4). Specifically, the right hippocampus presented stronger functional connectivity in the follow-up evaluation time compared to the pre-stimulation evaluation time in (1) a bilateral cortical region that encompasses the premotor cortex and extends to the supramarginal gyrus; (2) the left posterior insula; (3) ventral ACC bilaterally; (4) dorsal posterior cingulate cortex (PCC) extending to the precuneus and superior parietal cortex; and finally (5) the occipitotemporal cortex extending to the fusiform and lingual gyrus (see Fig. 4). Importantly, these regions have all been shown to be related with episodic memory and impairments on retrieval and encoding of episodic events [112]. No other effects were obtained for the cerebellum group.

Once more, the sham group also showed significant differences in the connectivity fingerprints of the hippocampi by evaluation time. Specifically, for both the right and left hippocampus (when comparing pre- and post-stimulation evaluation times), and for the right hippocampus (when comparing the pre-stimulation with the follow-up evaluation times), we found that a region around the precuneus (bilaterally), and extending to calcarine sulcus and occipitotemporal regions, showed increased connectivity. Moreover, the left hippocampus was also more strongly connected with certain parts of the right dorsal PCC, the right anterior PFC, and the right angular gyrus when comparing the pre- and the post-stimulation evaluation times, areas highly involved in episodic memory performance, when we compared pre- versus post-stimulation evaluation times (see Fig. 4). No other differences were significant for the sham group or any other group.

### Are there changes in structural connectivity after tDCS stimulation to the cerebellum?

**Fig. 5:**
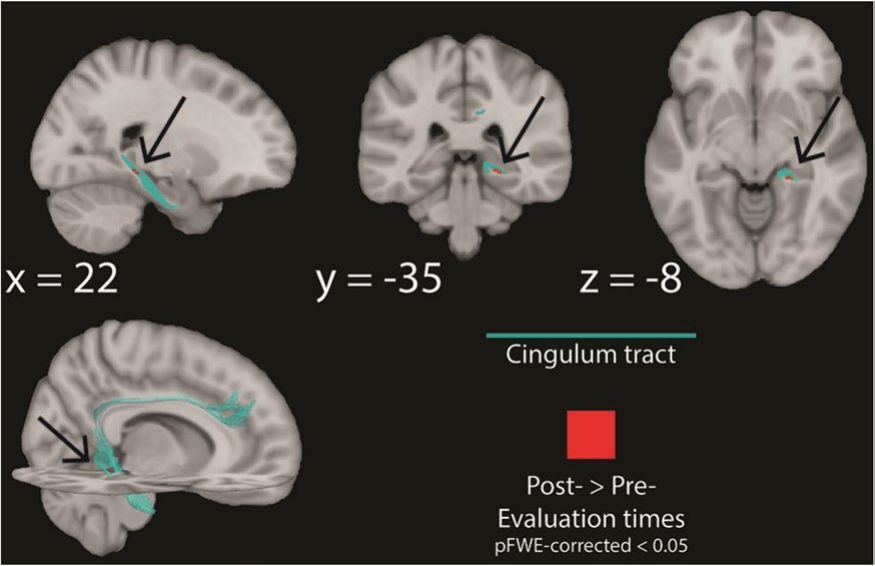
Structural connectivity effects of tDCS stimulation. Here, we show significant changes within white matter fiber bundles that have been shown to be important for episodic memory processes. Specifically, we show that for the right cerebellum anodal tDCS experimental group (only), there are significant improvements in diffusity (MA values) within the ventral part of the cingulum bundle at the post-stimulation evaluation time, when compared to the pre-stimulation evaluation time. Maps are thresholded at *p* < 0.05 FWE corrected

Finally, we focused on the effects of tDCS on episodic memory-related white matter tracts. Thus, we looked at whether and how typical white matter tracts associated with episodic memory — i.e., the fornix, the uncinate fasciculus, and the cingulum bundle [90, 95, 114] — were affected by tDCS plus cognitive training intervention. To do so, we compared the pre-stimulation versus the post-stimulation evaluation times, and the pre-stimulation versus the follow-up evaluation times for the different experimental groups on mode of anisotropy (MA) values [90] extracted using Tract-Based Spatial Statistics (TBSS). We used MA values because this diffusion measure can detect white matter alterations that other diffusion measures cannot [90]. As can be seen in Fig. 5, tDCS to the right cerebellum coupled with cognitive training lead to an increase of MA in the ventral part of the cingulum on the right hemisphere when comparing pre-stimulation vs post-stimulation evaluation times, suggesting higher linearity in the diffusion in the part of the cingulum that is closest to the hippocampus after stimulation of the cerebellum (*p*FWE-corrected < 0.05; no other effects for different white matter tracts, evaluation times, and stimulation conditions were significant). This shows, then, that diffusion in the ventral cingulum, the part of the cingulum that is most related with episodic memory [115], seems to increase predominantly in its typical orientation when comparing pre- and post-stimulation evaluation times after tDCS to the right cerebellum, suggesting more tuned structural connectivity in the cingulum around the hippocampus as a consequence of stimulating the cerebellum.

## Discussion

Here, we show that the cerebellum plays a causal role in episodic memory performance and aging-related decline, and that age-related episodic memory decline can be reduced in a long-lasting way with neurostimulation to the right cerebellum. Specifically, we show that under healthy aging, stimulating the right cerebellum enhances episodic memory both immediately and in a more stable way (at least 4 months after the intervention), while concomitantly bringing about a series of changes to the (functional and structural) connectivity fingerprints of the hippocampus and the remaining episodic memory network.

First, we showed that episodic memory performance in healthy elderly individuals — as measured by the FCSRT [44, 45] — was highly affected by our right cerebellum neurostimulation protocol. Participants in this experimental group (and not those in the remaining ones) were able, on average, to recall 3 more items, out of a total of 16, after the simulation program when compared to their pre-stimulation performance. This was true both right after the stimulation and in a follow-up visit 4 months after the stimulation program. That is, this memory performance enhancement greatly outlasts the stimulation period. Moreover, all individuals in the right cerebellum anodal tDCS experimental group showed memory performance improvements at the follow-up evaluation time, and 11 of those showed improvements immediately after the intervention. Notably, none of the individuals in the cerebellum group presented decrements in episodic memory performance after the simulation program.

Importantly, these effects on our primary outcome for the cerebellum group were accompanied by several tDCS-induced neural changes within episodic memory-related structures. Episodic memory depends heavily on relay loops between the hippocampus and other cortical and limbic structures [16], demonstrating the importance of connectivity in cognitive and neural processing [116–118]. This typically involves a hub of regions such as the PCC, the ACC, the ventromedial superior frontal gyrus, the medial PFC, the angular gyrus, the lingual gyrus, and the insula among other areas [8, 16, 24, 119] — most of which are also part of the default mode network [107, 120]. Connectivity between these areas and the hippocampus is a major predictor of episodic memory performance [120, 121], and it has been shown to be reduced both in normal aging [121] and in pathological aging (MCI and AD), where it correlates with episodic memory deficits [8, 122]. Importantly, most, if not all, of these areas showed increase connectivity with the hippocampus after tDCS to the right cerebellum (and not in any of the other experimental groups) and seem to be the neural hardware that supports the episodic memory enhancement that we observed.

Immediate effects of tDCS to the right cerebellum on the functional connectivity of the hippocampus were observed over a series of regions that are majorly involved in many of the processes that are central to episodic memory, and thus are important to sustain enhancements of episodic memory performance. Specifically, we showed increased connectivity of the hippocampus with regions engaged under episodic memory encoding, such as the ACC, that has been associated with deep encoding [104], and the use of self-referential information [104, 123] in episodic encoding (and retrieval) [105], as well as the medial and dorsolateral aspects of the PFC, that are responsible for deep episodic encoding of verbal material [12, 112]. Moreover, a region implicated in shallow encoding — the lingual gyrus [104, 113] — also showed increased functional connectivity with the hippocampus. We also showed heightened connectivity of the hippocampus with regions that have been implicated in retrieval of episodic details supported by rich contextual associations such as the left angular gyrus and the left superior frontal cortex [5, 105, 106, 108]. In fact, the role of the angular gyrus in retrieval of episodic details may relate to the fact that this area acts as an accumulator of multisensory episodic information used to inform retrieval decisions [5, 105], whereas the role of the more superior frontal area shown here may relate to selecting, monitoring, and maintaining retrieval cues and relevant information [5]. These functional changes were accompanied by changes in white matter diffusion of the ventral part of the cingulum bundle (for an example of effects of tDCS on white matter) [115]. Interestingly, this part of the cingulum has been associated with (verbal) episodic memory, shows impairments under MCI and AD, and connects most of the areas described above to the hippocampus [114]. Increased connectivity between the hippocampus and these areas immediately after the stimulation program may then relate to an overall enhancement of the major processes of encoding and retrieval that support the episodic memory enhancement observed for the cerebellum group.

More stable and long-lasting effects of the tDCS stimulation to the right cerebellum were observed in a set of regions that include some of those that show immediate effects — such as the ACC and superior frontal cortex — but also other areas such as the insula and the PCC — major players in episodic memory [26, 113, 124]. As a group, these areas support episodic retrieval and encoding aspects, as well as monitoring and maintaining retrieval cues [104–106, 108, 110, 113, 124, 125]. Moreover, the PCC has been shown to have an active role in memory consolidation [126], and to be a central area for the coordination of the episodic memory network in its efforts for encoding, consolidating, and retrieving of episodic events [127]. The focus on coordinating the different episodic memory regions and on memory consolidation may be supporting the long-lasting memory enhancements that were observed.

In the current study, no other experimental group showed episodic memory enhancements. However, if we adopt a more lenient approach (e.g., if we do not apply corrections for multiple comparisons), the DLPFC and sham experimental groups (groups where there were effective memory interventions; cf. wait-list) also show memory performance effects. These could be due to true memory enhancement from the cognitive training session (plus tDCS stimulation in the case of the DLPFC group), or it could be due to an effect of task and list training. In line with this, the DLPFC group showed no concomitant immediate or long-lasting neural effects that could support the potential memory effect. The sham group, however, showed neural changes after stimulation (both immediately and after 4 months) — this was particularly (and almost exclusively) true for the connectivity of the hippocampus with the precuneus and some occipitotemporal cortex regions. The precuneus is involved in recollection of past events and source memory [5, 105, 106, 108], and has been shown to be one of the major episodic areas benefitting from cognitive training [109]. Although we cannot adjudicate between whether the neural and behavioral effects observed for the sham group (and for the DLPFC group) were due to real memory enhancement, an effect of the cognitive stimulation protocol, or the effect of task and list training, we can certainly be sure that all these effects were magnitudes weaker than those obtained for the right cerebellum experimental group — both behaviorally and neurally, and immediately after the stimulation program and especially over a more stable period of time.

One important question relates to the mechanism by which the cerebellum impacts episodic memory and the functional neural network that supports it. It has been proposed that the cerebellum modulates behavior by regulating cognitive processes and maintaining proper function, as it does for motor performance — the universal cerebellar transform [28]. That is, it functions as an oscillation dampener [28–30] to ease the adaptation of the system to the particular context and the idiosyncrasies of the actions and/or processes being performed. This is applied, potentially equally, to all domains of function, due to the widespread connections the different parts of the cerebellum hold with association and limbic cortex [30]. Because of this, it has been proposed that there is a unique type of impairment following cerebellum lesions — dysmetria — with the specific type of dysmetria depending on the location of the lesion (i.e., the cortical targets of the bidirectional connections of that part of the cerebellum). In particular, Schmahmann and colleagues have extensively described patients with dysmetria of thought — the cerebellar cognitive affective syndrome [30]. This syndrome may lead to impairments in visual-spatial memory, planning and executive functioning, and verbal fluency, among other cognitive deficits [30]. These impairments would revolve around deficient connections between aspects of the cerebellum and some of the cerebral and limbic regions whose connectivity we have shown to be affected by our tDCS intervention to the right cerebellum (e.g., hippocampus, PCC, ACC, medial and dorsolateral PFC, superior frontal cortex). Thus, our stimulation program may have enhanced these regions and their connectivity fingerprints indirectly via the exchange of information between the cerebellum and cortical and limbic targets that typically sustains universal cerebellar transforms, and the maintenance of cognitive homeostasis that is ensured by the cerebellum, leading to a long-lasting enhancement of episodic memory abilities in healthy elderly individuals. Moreover, our tDCS stimulation of the cerebellum might have also affected the hippocampus directly [36, 39]. This stimulation effect would then percolate to the connectivity targets and episodic memory processing networks that relate to mnesic processing within the hippocampi. That is, whether directly or indirectly, episodic memory performance benefitted from the tDCS stimulation to the cerebellum both in terms of homeostatic effects on cognition and episodic memory processes implemented by heightened universal cerebellar transforms, as well as from enhanced processing of the hippocampi and subsequent memory gains.

Another outstanding question relates to how our results add to the field of non-invasive brain stimulation studies targeting the cerebellum, as well as those targeting episodic memory enhancement. Stimulation of the cerebellum has been shown to benefit cognitive performance [60], and specifically performance in (verbal) working memory tasks [60, 128, 129]. Moreover, neurostimulation of the cerebellum modulates connectivity of the DMN and the hippocampus to the cerebellum [33, 130]. However, neurostimulation interventions to the cerebellum on episodic memory retrieval are, to our knowledge, scarce, and have focused on dissociating episodic and semantic encoding [33]. In fact, most episodic memory neurostimulation-based interventions have targeted other regions. Unfortunately, the results of these interventions are not consistent [2, 131–133] — some studies show that neurostimulation of the DLPFC (predominantly the left) and parietal cortex leads to episodic memory improvement [134–137], whereas others fail to show facilitation effects [2, 134, 138, 139]. Thus, our study somewhat confirms that targeting DLPFC does not consistently enhance episodic memory performance, at least in a long-lasting way. Most importantly, though, we show long-term effects of tDCS stimulation of the cerebellum on episodic memory performance (compared with several control groups), and thus causally demonstrate that the cerebellum plays an important role in episodic memory performance and decline in normal aging, setting this region as a major target for age-related interventions.

Overall, our work then shows long-term improvements on episodic memory performance under healthy aging after neuromodulation of the right cerebellum, demonstrating the causal role of the cerebellum in high-level cognitive processes — and specifically in episodic memory. They also show that the cerebellum plays an important part in age-related decline. Although further work is needed, this work opens up the possibility of developing non-pharmacological interventions to ameliorate typical age-related cognitive frailty that induce long-lasting improvements that, at least, outlast the 4 months tested herein.

### Supplementary Information

Below is the link to the electronic supplementary material.

Supplementary file1 (DOCX 69 KB)

## Data Availability

All data and code will be available in https://osf.io.
