# Supplementary material for: The cerebellum is causally involved in episodic memory under aging
Source: GeroScience. 2023 Feb 7;45(4):2267–87. doi: 10.1007/s11357-023-00738-0 (PMC10651631; doi:10.1007/s11357-023-00738-0)
Supplement: Supplementary file 1 — Supplementary file1 (DOCX 69 KB) [file 11357_2023_738_MOESM1_ESM.docx]

Supplementary Materials for

The cerebellum is causally involved in episodic memory under aging

Almeida, J.^1,2*^, Martins, A.^1^, Amaral, L.^1,2^, Valério, D.^1,2^, Bukhari, Q.^1,2^, Schu, G.^1,2^, Nogueira, J.^2,3^, Spínola, M.^2,3,4^, Soleimani, G.^5^, Fernandes, F.^6^, Silva, A.R.^2,3^, Fregni, F.^7^, Simis, M.^8^, Simões, M.^2,3^, Peres, A.^1,2^

**Affiliations:**

^1^Proaction Lab, Faculdade de Psicologia e de Ciências da Educação, Universidade de Coimbra, Portugal

^2^CINEICC, Faculdade de Psicologia e de Ciências da Educação, Universidade de Coimbra, Portugal

^3^Psychological Assessment and Psychometrics Laboratory, Faculdade de Psicologia e de Ciências da Educação, Universidade de Coimbra, Portugal

^4^NOVA LINCS, University of Madeira, Caminho da Penteada, 9020-105 Funchal

^5^Biomedical Engineering Department, Amirkabir University of Technology (Tehran Polytechnic), Tehran, Iran.

^6^Grupo HPA Saúde, Alvor, Portugal

^7^Spaulding Neuromodulation Center, Department of Physical Medicine & Rehabilitation, Spaulding Rehabilitation Hospital and Massachusetts General Hospital, Harvard Medical School, Boston, MA

^8^Hospital das Clinicas HCFMUSP, Faculdade de Medicina, Universidade de São Paulo, São Paulo, Brazil

Correspondence to: [jorgecbalmeida@gmail.com](mailto:jorgecbalmeida@gmail.com)

**This PDF file includes:**

Materials and Methods

Tables S1

Materials and Methods

Participants

Fifty-six healthy elderly individuals (≥ 60 years old) were included in the study (34 female, 22 male; mean age = 68.5; Mean formal education years = 12.3; see Supplementary Table 1 for demographic information). Participants were recruited through broad-based advertisements in the community (e.g., flyers, websites, public talks) and referral institutions (e.g., senior universities, community health centers, nursing homes). All the participants were Portuguese native speakers, right-handed (as assessed by the Laterality tasks of the “Coimbra Neuropsychological Assessment Battery)(*71*), had no history of neuropsychiatric disorders (e.g., stroke, epilepsy, dementia, depression) or head injury, had no metallic implants, did not intake concurrent medication likely to affect cognition and had no history of alcohol and drug abuse. Written informed consent was obtained from all participants prior to the beginning of the study. Participants were each paid €50 upon completion of the study. This study was approved by the Ethics Committee of the Faculty of Psychology and Educational Sciences of the University of Coimbra, and performed following the ethical principles of research with human subjects.

Procedure

Pre-selection eligibility assessment was performed by phone and in-person before the study. Here we checked for major inclusion and exclusion criteria (those mentions above; e.g., medication, metal implants, laterality). Afterwards, participants went through a screening session. In this session, we assessed global cognitive function, using the Montreal Cognitive Assessment (MoCA)(*72*–*74*), and functional abilities using the Adults and Older Adults Functional Assessment Inventory(*75*, *76*). All participants performed above the cutoff for mild cognitive impairment, according to their age and educational level(*73*) (see supplementary table 2 for MoCA scores per group), and all except two showed functional disability scores in activities of daily living between 0 to 10%. Two participants revealed higher percentages of global functional disability (>14%), but these cognitive difficulties were not confirmed by MoCA scores and their physical limitations did not hamper their participation in the study. Finally, we also measured depression using the Geriatric Depression Scale (GDS)(*77*–*79*). None of the participants showed severe depressive symptomatology.

Upon inclusion in the study, participants were randomly assigned (1:1:1:1) to the following groups: 1) anodal tDCS to the left DLPFC plus cognitive training; 2) anodal tDCS to the right cerebellum plus cognitive training; 3) sham tDCS plus cognitive training; 4) wait list group. Participants allocated to the first three conditions were not informed as to what group they were allocated, neither whether they were receiving either active or sham tDCS stimulation. A minimal sample size of 11 participants per experimental group was determined taking in consideration that the primary outcome would be analyzed using a repeated measures ANOVA in a four-arm study with a significant level α of 5% with Bonferroni’s adjustment for multiple comparisons, an 80% power for a detection of a 1.5 increase in the z-score value of verbal episodic memory with a standard deviation of 1(*80*). Considering potential dropouts, the total number of participants per group in this study was set at 14 (56 in total). All participants completed 15 study sessions: the baseline assessment, 12 stimulation sessions, the post-intervention assessment and a 4-month follow-up. The 12 stimulation sessions were conducted on consecutive weekdays, at the same time of the day, and comprised 20 minutes of anodal tDCS (either to the left DLPFC or to the right cerebellum), followed by 1 hour of computer-based and pen-and-paper cognitive training tasks. Both the participant and the researcher administering cognitive training were blind to the experimental condition and only the researcher applying tDCS was aware of the allocation of participants per group. The assessment sessions (baseline, post-intervention and follow-up) included a neuropsychological assessment protocol, carried out by an external blinded rater, and a MRI, DTI and rest-fMRI session. After completion of the intervention sessions (session 12), participants in the tDCS training conditions were asked to guess whether they had received active or sham tDCS. Participants were unblinded to their experiment condition after the follow-up session.

Cognitive training tasks

The cognitive training program consisted of 12 sessions of computerized and pen-and-paper exercises focused on memory training, specifically verbal episodic memory. The training was organized in 6 sessions per modality (computer/pen-and-paper), administered in alternate order to increase the interest of the participants throughout the session. For the interactive computerized exercises, we used memory training tasks from the RehaCom cognitive rehabilitation software (Hasomed Inc, Magdeburg, Germany) that have been shown to enhance memory in healthy aging and mild cognitive impairment (namely its memory training modules; e.g., Memory for Words module and Physiognomic Memory)(*81*, *82*). Pen-and-paper tasks focused also on memory training exercises that included a working memory task, a semantic memory task, a face memory task and an autobiographical memory task taken from a Portuguese paper and pencil memory training program (Memo+)(*83*).

Before each exercise, participants were first given a verbal explanation of the tasks to be performed. In the computerized tasks, all participants began training at the beginner’s level of the RehaCom software. The training modules automatically adapted the training tasks to the user’s level of performance – according to whether the participant succeeded or failed the task, the difficulty levels were automatically adjusted to meet the participant’s ability. The same procedure was applied to the pen-and-paper tasks, where the difficulty levels were always adapted to each participant’s performance. All participants were trained on the same tasks for the same time, and the duration of each session was 1h (plus 20 minutes of tDCS).

tDCS montage

Anodal tDCS was applied daily for 12 consecutive weekdays in all experimental sessions using a TCT Stimulator Model 101 (Research Limited, Hong Kong, China). From the four intervention groups mentioned above, two received active tDCS and one received sham tDCS. In the active tDCS groups, electric stimulation was given continuously for 20 minutes at an intensity of 2mA. The anode electrode was either placed on the left DLPFC, over the F3 location according to the 10-20 EEG international system, or over the right cerebellar cortex, following the set up proposed by Pope and Mial(*84*) – 1cm under and 4cm lateral to the inion. In the sham condition, current was applied for 60 seconds only (30 seconds ram up and 30 seconds down), and the electrodes were placed as in the DLPFC montage. It should be noted that less than 3 minutes of tDCS induces no effects on cortical excitability (*85*) and using 60 seconds of stimulation is a reliable method of blinding, as it induces similar sensations on the scalp as real tDCS. For each montage, the reference electrode (cathode) was applied over the right deltoid muscle. The extra-cephalic reference was used to avoid the possible confounding effects that may be induced by two electrodes with opposite polarities. For all conditions, the anode and cathode rubber electrodes (5 x 5 cm, 25cm2) were placed inside a sponge soaked in saline solution and held in place by cloth straps with Velcro (see Figure 1B and C for tDCS montage and current flow maps). Throughout the duration of each session, the participants were accompanied by a researcher, who continuously monitored the current intensity and impedance. The tDCS device was placed by the researcher, while s/he was behind the participant, to preserve blinding. After every tDCS session, the stimulation sites were checked for side-effects and a questionnaire was filled out.

Neuropsychological Assessment

The neuropsychological assessment sessions were conducted before, immediately after and 4 months after the intervention to assess its long-term effects. Episodic memory performance was the primary outcome of this study. To assess episodic memory performance, we used the Portuguese version of the Free and Cued Selective Reminding Test (FCRST)(*39*, *40*). FCRST is an instrument that assesses verbal memory and learning through the presentation of a 16-word list memory test. There are three rehearsals of free and cued reminding (with 20 seconds of an interfering exercise between them) and a delay recall test (30 minutes after). Our outcome variable was exclusively the unguided delay recall test. A parallel version of FCRST was used in the post-intervention assessment session, in order to avoid learning and practice effects. The parallel version differed exclusively on the words presented, maintaining the number of words and respective categories.

Neuropsychological assessment included a series of other neuropsychological tests that were not considered primary outcomes and are not analyzed in the present study. These included the following instruments: 1) Subjective Memory Complaints (SMC)(*86*, *87*), used to characterize memory complaints; 2) Continuous Visual Memory Test (CVMT)(*88*, *89*), that evaluates visual memory ability; 3) Toulouse-Piéron Cancellation Test(*90*, *91*), a cancellation task that evaluates selective and sustained attention; 4) “Symbol Search” and “Digit Symbol-Coding” – Subtests of the Wechsler Adult Intelligence Scale (WAIS-III)(*92*, *93*), that measure information processing speed, visual perception, speed of processing and executive functioning; 5) Stroop(*94*), that assesses the ability to inhibit interference; 6) Semantic Verbal Fluency Test (SVF)(*95*–*97*), that evaluates the abilities of processing speed, language production and executive functions; and 7) World Health Organization Quality of Life-Older Adults Module (WHOQOL-OLD)(*98*–*100*), that assesses self-perceived quality of life. Average scores per group on these tests are presented in supplementary table 2.

MRI acquisition and preprocessing

Whole-brain fMRI data were collected with a 3-Tesla Siemens MAGNETOM trio MRI scanner (Siemens Healthineers, Erlangen, Germany) using a standard 12-channel head coil. High-resolution structural MRI data were acquired using a T1-weighted magnetization prepared rapid gradient echo (MPRAGE) sequence, which entailed the following parameters: 256 × 256 acquisition matrix, 256 mm field-of-view (FoV), voxel size of 1.0 × 1.0 × 1.0 mm3, flip angle (α) of 7º, bandwidth (BW) of 200 Hz/px, repetition time (TR) of 2530 ms and an echo time (TE) of 3.29 ms.  For resting-state fMRI data acquisition, participants were instructed to remain awake, laid supine, and with their eyes open, looking to a fixation cross, for 6 minutes. We used a T2*-weighted gradient echo-planar imaging (EPI) sequence with the following specifications: 64 × 64 acquisition matrix, 256 mmm FoV, flip angle of 90º, 33 interleaved slices, voxel size of 4.0 × 4.0 × 4.0 mm3, BW of 1562 Hz/px, TR of 2200 ms and TE of 30 ms. Finally, diffusion weighted images (DWI) were acquired using a single shot echo-planar sequence, with 69 diffusion directions, TR of 8900 ms and TE of 86 ms, diffusion weighting factor (b) of 1000 s/mm2, 70 slices with isotropic voxel resolution of 2 x 2 x 2 mm3 and 10 non-diffusion weighted (b = 0 s/mm2) volumes.

Both anatomical and functional data were pre-processed using fMRIPrep 20.1.1. This pipeline includes standard preprocessing steps, and its implementation aims to respond to the lack of an easy-usage workflow that ensures robustness independently of the data idiosyncrasies, and to increase consistency of fMRI results(*101*). For each of the 3 resting-state runs obtained per subject (one for each session: pre-stimulation evaluation time, post-stimulation evaluation time, and follow-up), the following preprocessing was performed. First, the five first volumes were skipped and a reference BOLD volume and its skull-stripped version were generated. Head-motion parameters with respect to the BOLD reference (transformation matrices, and six corresponding rotation and translation parameters) were estimated before any spatiotemporal filtering was employed using mcflirt (FSL 5.0.9)(*102*). BOLD runs were slice-time corrected using 3dTshift from AFNI 20160207(*103*) (RRID:SCR_005927). The BOLD reference was then co-registered to the T1-weighted image reference using bbregister from FreeSurfer (version 6.0.0)(*101*) which implements boundary-based registration(*104*). The BOLD time-series were resampled onto the MNI152NLin2009cAsym using the combined volumetric and surface-based (CVS) registration from Freesurfer (version 6.0.0)(*105*) and smoothed using SUSAN(*106*) with brightness threshold equal to 75% of the median brightness of the input image, FWHM of 6mm, and the Univalue Segment Assimilating Nucleus (USAN) was defined over the reference BOLD volume. Non-aggressive AROMA denoising (ICA-AROMA)(*107*) was performed on the registered functional data using the FilterRegressor function from Nipype (version 1.3.2)(*108*). Additionally, we calculate the CSF and WM average temporal series after AROMA-denoising (NiftiLabelsMasker - Nilearn version 0.6.2)(*109*) and regressed them out, alongside the six head-motion parameters using the TProject from Nipype (version 1.3.2)(*108*). Finally, we performed a second-order polynomial detrending and a temporal bandpass filtering of 0.009 and 9999 Hz.

Diffusion data was preprocessed using an in-house pipeline written in MATLAB R2019a that combined features of the FDT diffusion module from the FMRIB (Functional Magnetic Resonance Imaging of the Brain's diffusion toolbox) Software Library (FSL, version 6.0.4), and tools from the MRtrix3 software (<https://www.mrtrix.org/>; the codes are available at <https://github.com/maismemoria/preprocessing>). First, the acquired DWI data were converted from DICOM to NIFTI and inspected for visual quality. For each subject, images were denoised using the MP-PCA method(*110*) and non-weighted diffusion images (b0s) were used to generate a whole brain mask(*111*). Next, participant motion and eddy current distortions were corrected using the *eddy_openmp* program, which is part of the FSL FDT toolbox(*112*). Diffusion tensor was estimated via least square fitting and the mode of anisotropy (MA) measure was computed to characterize the diffusion displacement in the microstructure of white matter. The MA measure(*113*) is a real value defined in the interval between -1 and 1, and indicates how planar or linear is the tensor, respectively. A planar diffusion tensor is characterized to have two large eigenvalues and one small eigenvalue, whereas a linear diffusion tensor is characterized to have one large eigenvalue and two small eigenvalues. Studies have reported that the MA can detect white matter alterations that other diffusion measures cannot(*54*).

Data Analysis

For our primary outcome (i.e., episodic memory performance), a 4 (Experimental condition: Wait-list; Sham: left DLPFC; right Cerebellum) X 3 (Assessment times: Pre-stimulation evaluation time; post-stimulation evaluation time; 4-month follow-up) repeated measures ANOVA was calculated over the episodic memory performance (i.e., the delayed FCSRT measure) of our participants. Our main interest was the Experimental Condition *Assessment time interaction, and specifically the simple effects comparing for each group the pre- versus the post-stimulation evaluation time and the pre-stimulation evaluation time versus the 4-month follow-up. We used Bonferroni-corrected post-hoc t-tests to test for these comparisons.

In what regards our secondary outcomes (i.e., the MRI functional and structural connectivity) we used different analytical pipelines. For the functional connectivity analysis, we used two approaches: a region-wise strategy, where we defined Regions-of-Interest (ROI) and calculated functional connectivity between the hippocampi and a set of predefined ROIs, and a whole brain voxelwise approach where we calculated functional connectivity of the hippocampi with every voxel in the brain.

For the ROI analysis, we defined the ROIs to be used based on a Neurosynth meta-analysis map provided by the uniformity test (https://neurosynth.org/) using the keywords “episodic memory” (332 studies were included, FDR corrected < 0.01). The Episodic Memory Neurosynth map was resampled onto the MNI152NLin2009cAsym (isovoxel of 4mm) using the combined volumetric and surface-based (CVS) registration from Freesurfer (version 6.0.0)(*114*). Then we applied a simple cluster algorithm (first-neighbors contiguous voxels) in order to segregate the Neurosynth map in discrete regions. In each of the output clusters, we identified the voxel with the highest value, and using it as a seed of a region-growing algorithm, we used the highest 50 contiguous voxels (3.2 cm^3^) to fill in the ROI around the peak value. In total, we found 19 ROIs that, at least in part, overlap with the following anatomic regions: left hippocampus, right hippocampus, anterior cingulate cortex (ACC), ventromedial prefrontal cortex (PFC), left inferior frontal gyrus, right inferior frontal gyrus, left anterior insula, right anterior insula, left parahippocampal gyrus, right parahippocampal gyrus, dorsal posterior cingulate cortex (PCC), left ventral PCC, right ventral PCC, left precuneus, right precuneus, left intraparietal sulcus, right intraparietal sulcus, left angular gyrus, and right angular gyrus. After selecting the ROIs, for each of the three sessions, we calculate the functional connectivity (Pearson Correlation Fisher transformed) of the left and right hippocampus with all other selected regions producing a connectome with 35 unique pairs. Then, we tested the post-stimulation and follow-up connectomes against the pre-stimulation evaluation time, using a paired one-tail two-sample t-test. The results were corrected by the False Discovery Rate (FDR < 0.05)(*115*).

For the whole brain voxelwise analysis, we used the same left and right hippocampus ROIs defined in the ROI-to-ROI analysis as the seeds to calculate the seed-to-voxel connectivity. The BOLD volumes were gray-matter masked, ensuring that all non-zero voxels of all subjects' gray matter masks (automatically defined by fMRIPrep) were included. For each seed, we applied a general linear model using its average time series as the design matrix, resulting in 6 beta maps (two seeds and three sessions) per subject. Next, we compared whether there were differences in the beta maps of the post-stimulation and follow-up evaluation times compared to the pre-stimulation evaluation time. To test these hypotheses, we used the function Randomise two-sample paired t-test with 5000 permutations and corrected by threshold-free cluster enhancement (TFCE; FSL package)(*116*, *117*) and for multiple comparisons (*p*FWE-corrected < 0,05).

In the structural connectivity analysis, we examined three white matter tracts that have been previously reported to be associated with episodic memory, the fornix, the uncinate fasciculus and the cingulum bundle(*53*, *118*–*120*). The fornix is known to be associated with episodic memory performance in various neuropathological conditions(*53*). Similarly, degradations in the uncinate fasciculus have been reported to affect memory components(*121*). Lastly, it has been suggested that alterations in the cingulum bundle precede hippocampal atrophy influencing episodic memory performance in different syndromal stages(*122*, *123*). We estimated these tracts using the Tract-Based Spatial Statistics package (TBSS)(*124*). Briefly, the computed diffusion tensor maps were non linearly registered, aligning each subject and each measure map into a common space (FMRIB58_FA - 1x1x1 mm3). For each measure, we created average maps and their corresponding skeletons (thinned measured maps containing the centers of all white matter tracts common to a group of interest)(*124*). Then, voxel-wise statistical analysis, constrained by the ROIs in the white matter skeleton (JHU-DTI atlas)(*125*), was computed using Randomise Paired t-test with 5000 permutations using TFCE(*117*). Results were corrected for multiple comparisons (*p*FWE-corrected < 0.05).

Table S1.

Age and level of education by experimental group

|  |  | Age | Education |
| --- | --- | --- | --- |
| Experimental  Group | Wait-list | 68 (0.9) | 13 (1.3) |
|  | Sham | 69 (1.3) | 11 (1.4) |
|  | Left DLPFC | 68 (1) | 12 (1.3) |
|  | Right Cerebellum | 69 (1.7) | 13 (1.1) |
|  |  | *F* < 1 | *F* (3,52) = 147  *p* = 0.69 |

Average age and education level and SEM values
